# Investigating the effect of Shenmai injection on cardiac electrophysiology and calcium signaling using human-induced pluripotent stem cell-derived cardiomyocytes

**DOI:** 10.1016/j.bbrep.2022.101407

**Published:** 2022-12-26

**Authors:** Zhang Zhang, Yuxin Li, Meihui Yan, Tingting Yu, Xin Yuan, Sen Li

**Affiliations:** School of Life Sciences, Beijing University of Chinese Medicine, Beijing, China

**Keywords:** Calcium indicators, hiPSC-CMs, Shenmai injection, Traditional Chinese medicine, TCM, traditional Chinese medicine, TCMI, traditional Chinese medicine injections, hiPSC-CMs, human-induced pluripotent stem cell-derived cardiomyocytes, AP, action potentials, MEA, microelectrode array, FP, field potential, ECG, electrocardiogram, SMI, Shenmai injection, LC-MS, liquid chromatography-mass spectrometry, CCK-8, cell counting kit-8 assay, QTc, corrected QT, FWHM, full width at half maximum, CVDs, cardiovascular diseases, QoL, quality of life, ECC, excitation–contraction coupling, *I*_kr_, rapidly activating delayed rectifier potassium current, Polytetrafluoroethylene, PTFE

## Abstract

Traditional Chinese medicine injection (TCMI) refers to the use of modern technology to make Chinese patent medicines in injectable forms, which shorten the onset time of the traditional Chinese medicine (TCM). Although there have been clinical cases in which Shenmai injection (SMI) was used to treat cardiovascular diseases (CVDs), there are no pharmacological experiments that investigate the efficacy of the drug *in vitro* or the underlying mechanisms.

**Aim of the study:**

We aimed to systemically evaluate the efficacy and investigate the mechanisms of SMI in modulating electrophysiology and calcium (Ca^2+^) signaling using a microelectrode array (MEA) and a genetically encoded Ca^2+^ indicator, GCaMP6s, respectively, in human-induced pluripotent stem cell-derived cardiomyocytes (hiPSC-CMs).

**Materials and methods:**

A MEA system was employed to record field potentials (FPs) in hiPSC-CMs. The QT interval is corrected by the RR interval, the reciprocal of the beating rate. GCaMP6s was used to measure Ca^2+^ signaling in hiPSC-CMs. Meanwhile, the transcriptome changes in hiPSC-CMs treated with 2% SMI were examined using RNAseq. In addition, the ingredients of SMI were investigated using liquid chromatography-mass spectrometry (LC-MS).

**Results:**

It was found that 0.5%, 1%, and 2% (v/v) SMIs could increase corrected QT (QTc) but did not change other FP parameters. GCaMP6s was successfully applied to measure the chronic function of SMI. The full width at half maximum (FWHM), rise time, and decay time significantly decreased after treatment with SMI for 1 h and 24 h, whereas an increased Ca^2+^ transient frequency was observed.

**Conclusions:**

We first used the Ca^2+^ indicator to measure the chronic effects of TCM. We found that SMI treatment can modulate electrophysiology and calcium signaling and regulate oxidative phosphorylation, cardiac muscle contraction, and the cell cycle pathway in hiPSC-CMs.

## Highlights

• Shenmai injection regulates field potentials in cardiomyocytes.
• Shenmai injection regulates calcium signals in cardiomyocytes.
• Shenmai injection regulates the transcriptome of cardiomyocytes.

## 1 Introduction

Arrhythmia is a common cardiovascular disease (CVD) that manifests as an irregular heart rate [1]. According to the newly released European society of cardiology guidelines, existing treatments are still dominated by the more traditional four types of antiarrhythmic drugs, but these drugs still risk causing arrhythmia or heart failure [2]. Common drugs for arrhythmia treatment include β-blockers and Ca^2+^ channel blockers [3]. For instance, atenolol, bisoprolol, and metoprolol are well-known β-blockers. Although these drugs can slow the heart's rhythm, they have certain side effects, such as causing sleepiness [4] and affecting thyroid activity [5] and liver function [6,7]. For patients with less severe disease, seeking appropriate adjuvant therapy with lower risks may improve symptoms. With the progress of many clinical trials, the efficacy of traditional Chinese medicine (TCM) has gradually become noticed [8,9]. In recent years, the appearance of traditional Chinese medicine injection (TCMI) has accelerated the onset speed in the human body due to the method of administration [10]. Containing the ingredients of Hongshen (*Panax ginseng C.A. Mey.*) and Maidong (*Ophiopogon japonicus (Thunb.) Ker Gawl.*) [11], Shenmai injection (SMI) is generally used to treat CVDs, including arrythmia or other chronic diseases, in clinical practice [12,13]. However, the specific chemical composition and mechanism of action of SMI are still unclear [14].

Human-induced pluripotent stem cell-derived cardiomyocytes (hiPSC-CMs) are an *in vitro* human cardiomyocyte model that has been widely used to establish disease models and test drug effects [15]. Cardiac electrophysiology and calcium signaling are crucial for cardiomyocytes to fulfill their contractile function [16]. To noninvasively study cardiac electrophysiology, a microelectrode array (MEA) can be used to record the extracellular field potential (FP), which is closely related to the clinical electrocardiogram waveform [17]. Thus, MEAs have been employed in drug screening experiments [[18], [19], [20]]. Chemical dyes, such as Fluo-4-AM, have been widely used to measure intracellular Ca^2+^ signals, but it is difficult for these dyes to measure the long-term (e.g., 24-h) efficacy of drugs *in vitro* because of dye leakage and cytotoxicity [21,22]. GCaMP6s, a genetically encoded fluorescent Ca^2+^ indicator, can overcome these drawbacks and has been used for long-term recording of Ca^2+^ signals [23,24].

In this study, we utilized a MEA system to evaluate the efficacy of Shenmai injection (SMI) on cardiac electrophysiology. Meanwhile, we used a Ca^2+^ indicator (GCaMP6s) for the first time to test the instantaneous and long-term effects of SMI on Ca^2+^ signals in hiPSC-CMs [25]. In addition, RNAseq was employed to study the potential genes and cell signaling pathways affected by SMI. Liquid chromatography-mass spectrometry (LC-MS) was employed to detect the chemical components of SMI. Thus, we aimed to use multiple techniques to evaluate the efficacy of SMI and to investigate its underlying mechanisms.

## 2 Methods

### 2.1 Cells and reagents

HiPSC-CMs were purchased from HELP Regenerative Medicine Technology Co., Ltd. (Nanjing, China). From Peking Union Medical College (Beijing, China), 293T cells were obtained, and the cells were cultured as described previously [26]. SMI was obtained from Chiatai Qingchunbao Co., Ltd. (Zhejiang, China). The formula for Tyrode solution was as follows: 140 mM NaCl, 1 mM CaCl_2_, 5 mM KCl, and 10 mM d-glucose buffered with 10 mM HEPES at pH 7.4. Methanol and acetonitrile were purchased from Merck (Darmstadt, Germany). Formic acid was purchased from West Asia Reagent (Shandong, China).

### 2.2 Cell counting Kit-8 assay (CCK-8)

HiPSC-CMs were seeded in 96-well plates. Media containing 1%, 2%, 4%, and 8% (v/v) SMI were added to the plates and cultured in an incubator at 37 °C with 5% CO_2_ for 24 h. A microplate reader (SpectraMax i3X, Molecular Devices, CA) was utilized to read the absorbance, and the cell viability was calculated to determine the concentration of SMI that should be used in our subsequent experiments.

### 2.3 Lentivirus packaging

GCaMP6s plasmid has been constructed in previous experiments [25]. In a six-well plate precoated with poly-L lysine, 293T cells were seeded and transfected when the cell density reached 90%. Lentivirus particles were generated by co-transfecting 293T cells with packaging plasmid, envelope plasmid, and GCaMP6s expression plasmid. After 48 and 72 h, the supernatant lentiviral particles were collected, and green fluorescent protein (GFP) fluorescence was observed under an inverted fluorescence microscope (Axiocam 702 mono, Zeiss, Germany). After the cardiomyocytes were transduced with lentiviral particles for 72 h, the GFP fluorescence was observed.

### 2.4 MEA

Cells were seeded in the center of a MEA dish (Multi-Channel Systems (MCS) GmbH, Reutlingen, Germany), and the changes in FP in hiPSC-CMs were recorded after treatments of 0.25%, 0.5%, 1%, and 2% (v/v) SMI. Gradient dosing was applied, and the results were analyzed using Cardio2D + software (MCS GmbH, Reutlingen, Germany).

### 2.5 Ca^2+^ imaging

HiPSC-CMs were seeded with a density of 1 × 10^4^ on confocal dishes coated with a plating solution. After 24 h, they were replaced with a cardiomyocyte culture medium, and the medium was changed every other day. The cells were transduced with GCaMP6s lentiviral particles, and a confocal microscope (FV3000, Olympus, Japan) was used to capture Ca^2+^ fluorescence in the cytoplasm. Tyrode solution was used to wash the cells before Ca^2+^ imaging. Intracellular free Ca^2+^ could bind to GCaMP6s in the hiPSC-CMs, and Ca^2+^ transients were recorded after SMI treatment for 5 min, 1 h, and 24 h. Peakcaller [27] was used to analyze Ca^2+^ signal-related parameters.

### 2.6 RNAseq

The effect of SMI on the gene expression of hiPSC-CMs was analyzed using the intelligent analysis platform of BGI (Hubei, China). The general process was to first perform quality control on the RNA collected from cell lysis, including the RNA concentration and purity (A260/A280). Reverse transcription was performed if the RNA met the standards, and a library was established. The quality of the constructed library was checked, and subsequent sequencing was performed. The data obtained by sequencing were called raw reads and were also quality-controlled to determine whether they were suitable for subsequent analysis. The data obtained in this step were called clean reads. Step-by-step comparison, quantification, difference analysis, and cluster analysis were then carried out. The gene expression level was quantified by the number of fragments per thousand base fragments (FPKM). Differentially expressed genes (DEGs) were defined as FPKM ≥2, and the false discovery rate (FDR) was ≤0.001.

### 2.7 LC-MS

LC-MS was performed on an ultra-high-performance liquid chromatograph system (Thermo Vanquish UHPLC, Thermo Fisher Scientific, USA). The general process was to centrifuge SMI at 4 °C and 12,000 rpm for 10 min, and the supernatant was then aspirated and diluted 2–100 times. Afterwards, 10 μL of internal standard (100 μg/mL) was added and passed through a 0.22 μm polytetrafluoroethylene filter for onboard testing. This was followed by metabolite quantitative analysis, that is, calculating the peak area of the metabolite to obtain the relative concentration and percentage of the metabolite in the sample. An analysis platform (Ultimate 3000LC, Q Exactive HF, Thermo, USA) and chromatographic column C18 (Zorbax Eclipse C18 (1.8 μm × 2.1 × 100 mm) were then employed for analysis. The mobile phase was composed of A (H2O + 0.1% formic acid) and B (acetonitrile). The flow rate was 0.3 mL/min and the column temperature was 30 °C, while the injection volume was 2 μL and the autosampler temperature was 4 °C. The mobile phase gradient elution program was 5% B (0–2 min), 30% B (2–6 min, linear gradient), 30% B (6–7 min), 78% B (7–12 min, linear gradient), 78% B (12–14 min), 95% B (14–17 min, linear gradient), 95% B (17–20 min), 5% B (20–21 min, linear gradient), and 5% B (21–25 min).

### 2.8 Statistical analysis

SPSS was used for the statistical analysis. For data passing the normality and homogeneity tests, a one-way analysis of variance (ANOVA) test was used. Otherwise, a non-parametric test was used. If the data did not conform to a normal distribution, the Kruskal–Wallis test was employed. *P* < 0.05 indicated a significant difference. All data results were expressed as mean ± standard error.

## 3 Results

**Fig. 1:**
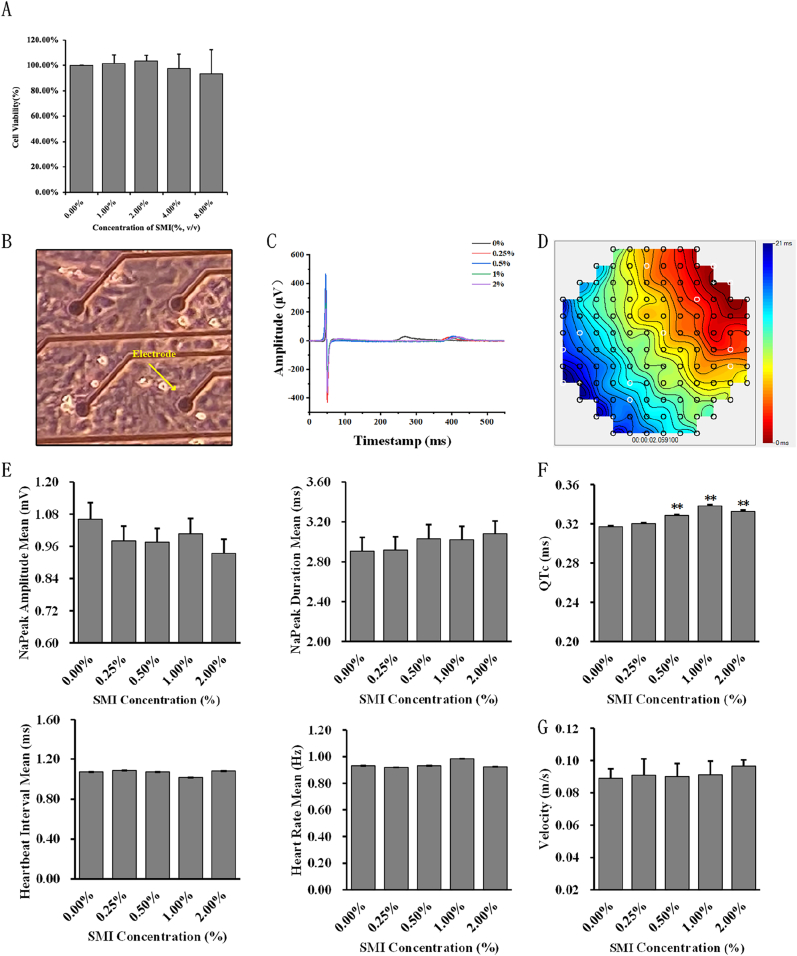
The effects of 1%, 2%, 4%, and 8% (v/v) SMI on hiPSC-CMs. (A) Increased concentrations of SMI did not show toxicity to hiPSC-CMs. (B) A representative diagram of the cells on the MEA. The yellow arrow points to the electrode. (C) Representative curves of field potentials. (D) A representative isochrone diagram of the conduction of electrical signals on the MEA. (E) Changes in electrical signal-related parameters with various SMI concentrations. (F) Changes in QTc with various SMI concentrations. (G) Changes in the conduction velocity of field potential propagation with various SMI concentrations. **P < 0.01. (For interpretation of the references to colour in this figure legend, the reader is referred to the Web version of this article.)

The effect of SMI on the cardiac functions of hiPSC-CMs has not yet been reported, and most *in vitro* cell experiments are based on H9C2 cells or rat cardiomyocytes [[28], [29], [30]]. Thus, the effective and non-toxic concentrations were first investigated using CCK-8. The CCK-8 results indicated that 1%, 2%, 4%, and 8% (v/v) SMI were not toxic to hiPSC-CMs (Fig. 1A). We next tested the potential effects of SMI on the electrophysiology of hiPSC-CMs using a MEA system (Fig. 1B–D). When 0.25%, 0.5%, 1%, and 2% (v/v) SMIs were added to hiPSC-CMs, no significant change was observed in field potential NaPeak amplitude, NaPeak duration, heartbeat interval, or beating rate (Fig. 1E). However, prolongation of QT could be observed when the concentration of SMI reached 0.5%, 1%, and 2% (Fig. 1F). Combining the results of the CCK-8 and MEA, as well as the concentrations of SMI reported in the existing studies, 2% SMI was employed to investigate the effect of SMI on calcium signaling in hiPSC-CMs.

**Fig. 2:**
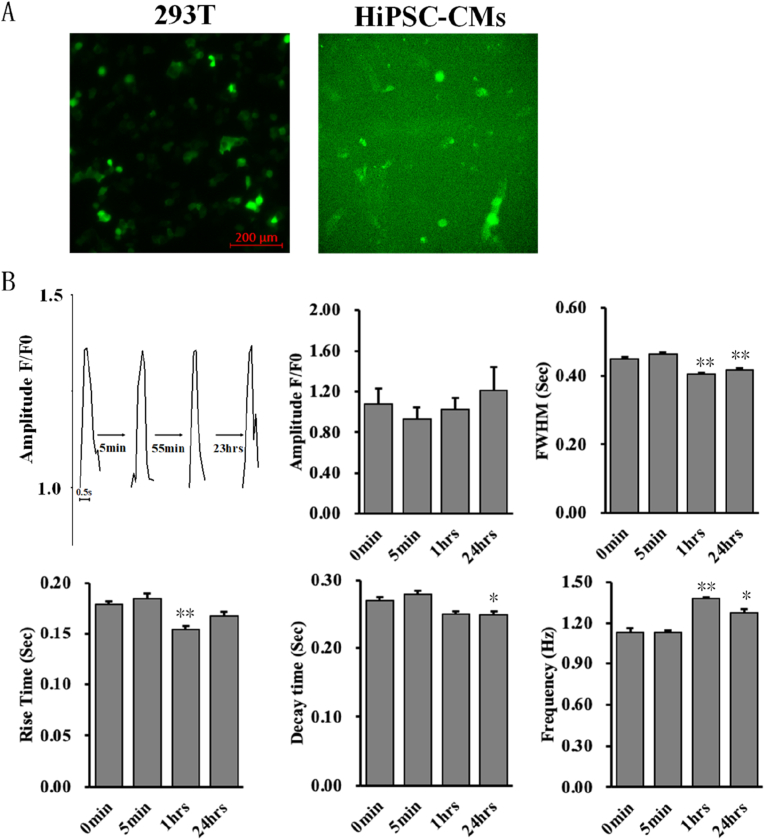
Ca^2+^ signaling in hiPSC-CMs recorded using GCaMP6s through lentiviral transduction. (A) The upper left picture represents the GFP fluorescence after GCaMP6s plasmids were transfected with 293T; the upper right picture represents the fluorescence after GCaMP6s protein was expressed and bound to free Ca^2+^ in hiPSC-CMs. (B) The alterations of Ca^2+^ transient-related parameters before and after the addition of 2% (v/v) SMI. The upper left represents a representative curve of the amplitude of Ca^2+^ transient at different times. *P < 0.05, **P < 0.01.

After transducing hiPSC-CMs with GCaMP6s lentiviral particles for 72 h, GFP fluorescence was observed under an inverted fluorescent microscope (Fig. 2A), and these cells were subsequently used to investigate the effect of SMI on cardiac Ca^2+^ signaling**.** We recorded Ca^2+^ signals 5 min, 1 h, and 24 h after SMI treatment, and the results showed that 2% (v/v) SMI did not change the Ca^2+^ transient-related parameters at 5 min (Fig. 2B). However, SMI significantly reduced the full width at half maximum (FWHM) and increased the frequency of Ca^2+^ transients after 1 h and 24 h of treatment. Moreover, SMI significantly reduced the rise time and decay time after 1 h and 24 h of treatment, respectively (Fig. 2B).

**Fig. 3:**
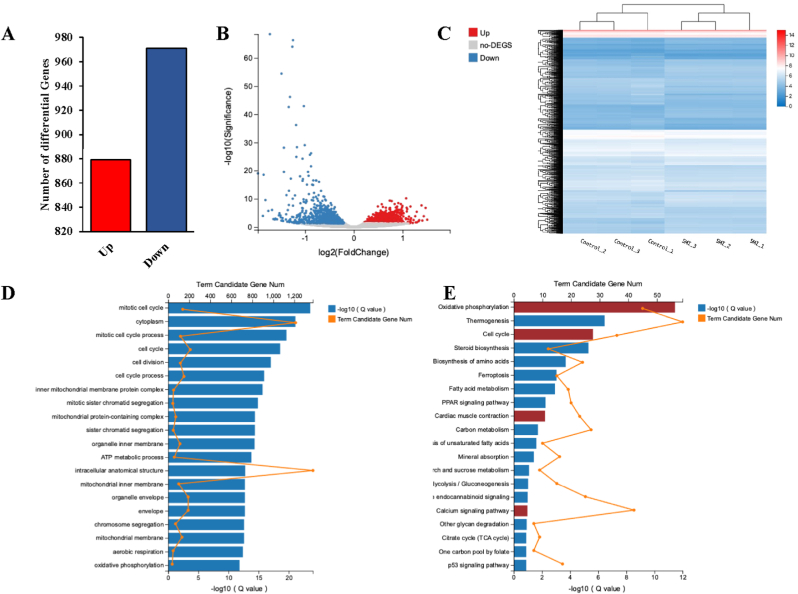
Changes in the gene expression of hiPSC-CMs induced by SMI 2% (v/v). (A) SMI treatment changed the expression of the genes. Red represents upregulated genes, and blue represents downregulated genes. (B) Scatter plot of differentially expressed genes. (C) A heatmap formed by clustering the FPKM values of differentially expressed genes for each comparison group. (D) A bar graph of the enrichment degrees of GO terms. (E) A bar graph of the enrichment degree of the KEGG pathway. (For interpretation of the references to colour in this figure legend, the reader is referred to the Web version of this article.)

RNAseq results indicated that, compared to the control group, 2% (v/v) SMI upregulated 879 genes and downregulated 971 genes (Fig. 3A–C). Differentially expressed genes were significantly enriched in oxidative phosphorylation, cardiac muscle contraction, and cell cycle pathways (Fig. 3D and E). To analyze the active ingredients of SMI, LC-MS was employed [31]. The total ion current (TIC) chromatogram is presented in Supplementary Figs. 1 and 82 components were detected. These components were identified mainly as organic oxygen compounds, pregnenolone lipids, carboxylic acids, and their derivatives. Among these, the percentage of D-(+)-maltose substance was the largest, followed by that of valtrate, which reached 41.4% and 3.6%, respectively. The retention times for D-(+)-maltose and valtrate were 0.78 and 9.10 min, and their molecular weights were 342.1 and 422.2, respectively. Detailed information is shown in Supplementary Table 1.

## 4 Discussion

Contraction, the most important function of the heart, is a consequence of cardiac electrophysiology and calcium signaling [32]. First, the plasma membrane of the cardiomyocyte is depolarized during the action potentials induced by electrical stimulation, which activates the influx of extracellular Ca^2+^ through voltage-dependent Ca^2+^ channels. The Ca^2+^ further activates ryanodine receptors (RyRs) on the membrane of the sarcoplasmic reticulum (SR), which triggers Ca^2+^ release from the SR into the cytoplasm. This process is known as calcium-induced calcium release [32]. Finally, these Ca^2+^ bind to contractile proteins, such as troponin, and cause contraction of the cardiomyocyte. The conversion of the AP into a contractile response is called excitation–contraction coupling [32]. The role of SMI in improving myocardial function and treating heart disease has been investigated by several researchers. For example, Zhang et al. found that SMI could inhibit myocardial autophagy and attenuate myocardial damage caused by doxorubicin [33], and Chen et al. found that SMI improved chronic heart failure [34]. Due to the importance of electrical and Ca^2+^ signals to cardiac contractile function, our study was conceptualized to investigate the role of SMI in modulating electrophysiology and calcium signaling in hiPSC-CMs, an *in vitro* human cardiomyocyte model.

In existing studies, the range of SMI concentrations used is around 0.25%–5%, but most of the cells under investigation are H9C2. In our study, we tested the effect of SMI with a wider range of concentrations (up to 8%) on the viability of hiPSC-CMs using a CCK-8 assay and found that it did not significantly alter cell viability. Therefore, the minimum effective concentrations of SMI of various doses below 2% were used in field potential recordings. As reflected by the parameters of the field potentials, QT prolongation was observed, which is generally believed to be associated with an increased risk of arrhythmia. However, it is worth noting that the concentrations of SMI used in this study are still higher than the clinical dosage. Abnormal Ca^2+^ handling can cause instability in AP duration by affecting the repolarization of hiPSC-CMs, but the results of our Ca^2+^ imaging experiments indicated that SMI did not alter the Ca^2+^ transients of hiPSC-CMs in short-term (i.e., 5-min) treatment, suggesting that SMI does not cause changes in the QT interval by altering calcium handling. The underlying mechanisms, especially the potential effects of SMI on the activities of various ion channels, deserve further investigation.

Because of the limited effects of short-term SMI treatment, we, for the first time, used a genetically encoded Ca^2+^ indicator (GCaMP6s) instead of chemical dyes to study the long-term effect of TCM on Ca^2+^ signaling in cardiomyocytes. GCaMP6s is not likely to leak out of cells compared to chemical dyes, such as Fluo-4 [35]. Moreover, although the signal-to-noise ratio of GCaMP6s is not as high as that of Fluo-4, it can be stably expressed in cardiomyocytes [36]. Thus, genetically encoded Ca^2+^ indicators have been widely used in the literature. For example, Chen et al. used GCaMP6 to measure the long-term activity of neuronal cells [37], and Pahlavan et al. used FKBP-GCaMP6, a modified Ca^2+^-sensing protein, as a molecular probe in ventricular myocytes [38]. Therefore, it is promising to use these Ca^2+^ indicators to study the long-term effects of TCM on Ca^2+^ signals, especially when considering the relatively slow onset of action of TCM. We then used GCaMP6s to study the Ca^2+^ signals in hiPSC-CMs treated with SMIs for a longer period (i.e., 1 h and 24 h), and our results showed that the FWHMs, rise times, and decay times of Ca^2+^ transients were significantly decreased, whereas Ca^2+^ transient frequency was significantly elevated, when the incubation time of the SMI was increased. According to one report, SMI has been reported to enhance energy metabolism while slightly increasing heart rate in patients with heart failure [39], which may also lead to a shortening of Ca^2+^ transient duration. As an indicator of Ca^2+^ transient duration, FWHM is correlated with the total amount of Ca^2+^ mobilized during one contraction cycle, which is quantified by the area under the spike of the calcium transient waveform. Thus, long-term SMI treatment reduces the lifespan of Ca^2+^ transients and promotes heartbeat pulsation.

Short-term drug treatment may alter protein activity by, for example, inhibiting calcium channels and phosphorylating receptor proteins, and long-term drug treatment may affect the transcriptome. Thus, we further investigated the effect of 2% SMI on hiPSC-CMs transcriptome using RNAseq. As enriched in KEGG, the signaling pathways related to oxidative phosphorylation and cardiac muscle contraction were affected by SMI. This is consistent with the literature, which indicates that SMI can improve energy metabolism and enhance myocardial contraction and myocardial function. For example, SMI combined with conventional treatment improved energy metabolism in patients with heart failure in a clinical trial [39], and SMI enhanced the cardiac contractility of patients with arrhythmia [40]. Interestingly, the cell cycle pathway has also been a target of SMI. The hiPSC-CMs used in this study were relatively immature cardiomyocytes. It has been shown that Ca^2+^ plays an important role in regulating cardiomyocyte maturation during early cardiomyocyte development [41]. It can be speculated that SMI may affect the cell cycle pathway and thus promote cardiomyocyte maturation, leading to increased activity in Ca^2+^ channels and receptors. This is consistent with the results that Ca^2+^ transients were altered by SMI treatment for 24 h in hiPSC-CMs. Due to the complex composition of SMI, we dissected its composition using LC-MS, and the compounds identified in SMI have recently been reported to target electrical and calcium signals. For example, ginsenoside has been reported to modulate voltage-gated ion channels by activating Na^+^/K^+^ channels [42] and to inhibit the overactivation of RyRs to prevent calcium leakage [43]. These findings corroborate that ginsenoside is one of the active components of SMI that affects hiPSC-CMs, and the role of these small molecules in modulating cardiac electrophysiology and calcium signaling warrants further investigation.

This study has several limitations. First, we only measured the efficacy of SMI at the cellular level, and animal models need to be established in subsequent studies to reflect the efficacy of SMI on changes in heart rate and myocardial function. Second, this article only evaluated the efficacy of SMI based on electrical and Ca^2+^ signals, and contractility could also be studied further [44]. In addition, the specific signaling pathways targeted by SMI have not been well elucidated. In the future, this should be studied by silencing the expression of key ion channel proteins, such as LTCC and RyRs, and by using ion channel blockers or activators. In addition, patch clamp techniques should be used to study the specific ion channels affected. Finally, this article only discussed the chemical components of SMI, and studying the effects of the major chemical components of SMI on myocardial function may lead to a better understanding of the mechanism of action of SMI.

## 5 Conclusion

We used a MEA system and a genetically encoded Ca^2+^ indicator (GCaMP6s) to study the efficacy of TCM, concluding that the therapeutic effects of SMI might be closely related to its ability to regulate electrical and Ca^2+^ signals. Multiple pathways are involved, such as the cardiac muscle contraction pathway.

## Authors’ contributions

SL designed the study. ZZ and YXL performed the experiments, analyzed the data, prepared the figures, and drafted the manuscript. MHY, TTY and XY critically reviewed the manuscript. All authors read and approved the final manuscript.

## Funding

This study is supported by the 10.13039/501100001809National Natural Science Foundation of China (Grant No. 81973698 and 81703942), Young Elite Scientists Sponsorship Program by CACM (Grant No. 2019-QNRC2-B08), Science Fund for Distinguished Young Scholars in BUCM (Grant No. BUCM-2019-JCRC004) and 10.13039/501100004846BUCM research start-up fund (to SL).

## Declaration of competing interest

All authors report no conflicts of interest.
