# Supplementary material for: Investigating the effect of Shenmai injection on cardiac electrophysiology and calcium signaling using human-induced pluripotent stem cell-derived cardiomyocytes
Source: Biochem Biophys Rep. 2022 Dec 26;33:101407. doi: 10.1016/j.bbrep.2022.101407 (PMC9803683; doi:10.1016/j.bbrep.2022.101407)
Supplement: Multimedia component 1 [file mmc1.docx]

**** Supplementary Figure 1. The components of SMI detected using LC-MS. The top of the figure shows the peaks detected in the positive ion mode, and the bottom of the figure shows the peaks detected in the negative ion mode.

Supplementary table 1. Compounds in SMI detected by LC-MS.

| **Name** | **Formula** | **CAS_num** | **Class** | **Annotation MW** | **Calc. MW** | **RT [min]** | **Relative concentrations of each components in SMI（μg/mL）** | **Relative percentage of components in SMI（%）** |
| --- | --- | --- | --- | --- | --- | --- | --- | --- |
| 4-methylpyridine-2,3-diamine | C_6_H_9_N_3_ | 53929-59-2 | Pyridines and derivatives | 123.07965 | 123.07968 | 18.744 | 101.404648 | 4.475% |
| Valtrate | C_22_H_30_O_8_ | 18296-44-1 | Prenol lipids | 422.19407 | 422.1942 | 9.103 | 977.785566 | 3.633% |
| DL-Arginine | C_6_H_14_N_4_O_2_ | 7200-25-1 | Carboxylic acids and derivatives | 174.11168 | 174.11176 | 0.726 | 69.297056 | 3.058% |
| Diethylpyrocarbonate | C_6_H_10_O_5_ | 1609-47-8 | Organic carbonic acids and derivatives | 162.05282 | 162.05274 | 0.782 | 58.4914057 | 2.581% |
| Asarone | C_12_H_16_O_3_ | 2883-98-9 | Phenol ethers | 208.10994 | 208.11002 | 9.085 | 35.7455881 | 1.577% |
| Choline | C_5_H_13_NO | 62-49-7 | Organonitrogen compounds | 103.09971 | 103.10011 | 0.748 | 33.1509515 | 1.463% |
| Adenosine | C_10_H_13_N_5_O_4_ | 58-61-7 | Purine nucleosides | 267.09675 | 267.09667 | 1.224 | 26.3372559 | 1.162% |
| Pyrogallol | C_6_H_6_O_3_ | 87-66-1 | Phenols | 126.03169 | 126.03181 | 0.795 | 21.7792288 | 0.961% |
| D-(+)-Proline | C_5_H_9_NO_2_ | 344-25-2 | Carboxylic acids and derivatives | 115.06333 | 115.06356 | 0.797 | 20.526507 | 0.906% |
| Roburic acid | C_30_H_48_O_2_ | 6812-81-3 | Prenol lipids | 440.36543 | 440.36547 | 7.427 | 16.343947 | 0.721% |
| 1-hexadecyl-2-amino-2-deoxy-sn-glycerol | C_19_H_41_NO_2_ | 136770-76-8 | Organonitrogen compounds | 315.31373 | 315.31365 | 11.339 | 16.0317046 | 0.707% |
| L-Pyroglutamic acid | C_5_H_7_NO_3_ | 98-79-3 | Carboxylic acids and derivatives | 129.04259 | 129.04276 | 0.939 | 15.8222981 | 0.698% |
| Falcarindiol | C_17_H_24_O_2_ | 55297-87-5 | Fatty Acyls | 260.17763 | 260.17763 | 11.665 | 13.7595598 | 0.607% |
| Spirodiclofen | C_21_H_24_Cl_2_O_4_ | 148477-71-8 | Benzene and substituted derivatives | 410.10516 | 410.10343 | 0.752 | 158.285883 | 0.588% |
| Glochidone | C_30_H_46_O | 6610-55-5 | Prenol lipids | 422.35487 | 422.35478 | 9.278 | 13.0224612 | 0.575% |
| Ginsenoside Rh4 | C_36_H_60_O_8_ | 174721-08-5 | Prenol lipids | 620.42882 | 620.42901 | 7.431 | 12.2631875 | 0.541% |
| Camellioside B | C_55_H_86_O_25_ | 378244-83-8 | Prenol lipids | 1146.54582 | 1146.55002 | 9.343 | 11.2704067 | 0.497% |
| Furaltadone | C_13_H_16_N_4_O_6_ | 139-91-3 | Furans | 324.10698 | 324.10548 | 0.85 | 133.639304 | 0.497% |
| Curcumene | C_15_H_22_ | 644-30-4 | Prenol lipids | 202.17215 | 202.17216 | 7.428 | 11.1173997 | 0.491% |
| Lupenone | C_30_H_48_O | 1617-70-5 | Prenol lipids | 424.37052 | 424.37035 | 9.589 | 10.4016094 | 0.459% |
| N-Boc-N'-Fmoc-diaminoacetic acid | C_22_H_24_N_2_O_6_ | 176039-39-7 | Fluorenes | 412.16344 | 412.16539 | 9.104 | 88.2197081 | 0.328% |
| L-Norleucine | C_6_H_13_NO_2_ | 327-57-1 | Carboxylic acids and derivatives | 131.09463 | 131.09474 | 1.367 | 7.38298568 | 0.326% |
| Pyroglutamic acid | C_5_H_7_NO_3_ | 98-79-3 | Carboxylic acids and derivatives | 129.04259 | 129.04266 | 0.762 | 5.86256449 | 0.259% |
| L-Phenylalanine | C_9_H_11_NO_2_ | 63-91-2 | Carboxylic acids and derivatives | 165.07898 | 165.07907 | 2.245 | 5.68511487 | 0.251% |
| Indoline | C_8_H_9_N | 496-15-1 | Indoles and derivatives | 119.0735 | 119.07374 | 2.243 | 5.61916907 | 0.248% |
| 20(R)-Notoginsenoside | C_41_H_70_O_13_ | 948046-15-9 | Prenol lipids | 770.48164 | 770.48193 | 9.494 | 51.3715407 | 0.191% |
| Citric acid | C_6_H_8_O_7_ | 77-92-9 | Carboxylic acids and derivatives | 192.027 | 192.02623 | 1.117 | 50.881861 | 0.189% |
| Adipic acid | C_6_H_10_O_4_ | 124-04-9 | Fatty Acyls | 146.05791 | 146.05816 | 18.225 | 48.0755927 | 0.179% |
| Trans-3-Indoleacrylic acid | C_11_H_9_NO_2_ | 1204-06-4 | Indoles and derivatives | 187.06333 | 187.06337 | 4.551 | 4.03344957 | 0.178% |
| N-Acetyl-L-aspartic acid | C_6_H_9_NO_5_ | 997-55-7 | Carboxylic acids and derivatives | 175.04807 | 175.04843 | 19.714 | 44.8413713 | 0.167% |
| Cis,cis-Muconic acid | C_6_H_6_O_4_ | 1119-72-8 | Fatty Acyls | 142.02661 | 142.02691 | 19.336 | 43.5966549 | 0.162% |
| Guanine | C_5_H_5_N_5_O | 73-40-5 | Imidazopyrimidines | 151.04941 | 151.0495 | 1.323 | 3.60300313 | 0.159% |
| 5-(Hydroxymethyl)-3-isoxazolecarboxylic acid | C_5_H_5_NO_4_ | 123770-62-7 | Azoles | 143.02186 | 143.02215 | 19.696 | 41.6702592 | 0.155% |
| Adenine | C_5_H_5_N_5_ | 73-24-5 | Imidazopyrimidines | 135.0545 | 135.0546 | 1.226 | 3.33097422 | 0.147% |
| Crotonoside | C_10_H_13_N_5_O_5_ | 1818-71-9 | Purine nucleosides | 283.09167 | 283.09163 | 1.323 | 3.07743339 | 0.136% |
| Cytarabine | C_9_H_13_N_3_O_5_ | 147-94-4 | Pyrimidine nucleosides | 243.08552 | 243.08589 | 0.82 | 2.81144705 | 0.124% |
| Indole | C_8_H_7_N | 120-72-9 | Indoles and derivatives | 117.05785 | 117.0581 | 4.517 | 2.67881672 | 0.118% |
| 3-SUCCINIMIDOPROPIONIC ACID | C_7_H_9_NO_4_ | 5724-76-5 | Carboxylic acids and derivatives | 171.05316 | 171.05356 | 19.355 | 31.5211311 | 0.117% |
| Betulin | C_30_H_50_O_2_ | 473-98-3 | Prenol lipids | 442.38108 | 442.38097 | 9.341 | 2.65201796 | 0.117% |
| Mitomycin | C_15_H_18_N_4_O_5_ | 50-07-7 | Indoles and derivatives | 334.12772 | 334.12647 | 5.777 | 31.4495736 | 0.117% |
| Succinic acid | C_4_H_6_O_4_ | 110-15-6 | Carboxylic acids and derivatives | 118.02661 | 118.02679 | 18.784 | 30.1753842 | 0.112% |
| L-Tyrosine | C_9_H_11_NO_3_ | 60-18-4 | Carboxylic acids and derivatives | 181.07389 | 181.07401 | 1.23 | 2.49107616 | 0.110% |
| Glutaric acid | C_5_H_8_O_4_ | 110-94-1 | Carboxylic acids and derivatives | 132.04226 | 132.04217 | 0.767 | 29.2242532 | 0.109% |
| D-(+)-Pipecolinic acid | C_6_H_11_NO_2_ | 1723-00-8 | Carboxylic acids and derivatives | 129.07898 | 129.0791 | 0.837 | 2.17355463 | 0.096% |
| LPE 18:2 | C_23_H_44_NO_7_P | 85046-18-0 | Glycerophospholipids | 477.28554 | 477.28563 | 12.257 | 2.08380883 | 0.092% |
| Clavulanic acid | C_8_H_9_NO_5_ | 58001-44-8 | Carboxylic acids and derivatives | 199.04807 | 199.04863 | 19.714 | 24.6968387 | 0.092% |
| Pseudouridine | C_9_H_12_N_2_O_6_ | 1445-07-4 | Nucleoside and nucleotide analogues | 244.06954 | 244.06927 | 1.132 | 24.6213152 | 0.091% |
| Trans-Cinnamaldehyde | C_9_H_8_O | 104-55-2 | Cinnamaldehydes | 132.05751 | 132.05768 | 8.359 | 1.93440768 | 0.085% |
| PC(16:0/0:0) | C_24_H_50_NO_7_P | 17364-16-8 | Glycerophospholipids | 495.33249 | 495.33241 | 12.871 | 1.92915527 | 0.085% |
| Cytosine | C_4_H_5_N_3_O | 71-30-7 | Diazines | 111.04326 | 111.04348 | 19.218 | 1.62082725 | 0.072% |
| Ginsenoside Rh3 | C_36_H_60_O_7_ | 105558-26-7 | Prenol lipids | 604.4339 | 604.43395 | 9.869 | 1.40529373 | 0.062% |
| Dodecyl sulfate | C_12_H_26_O_4_S | 151-41-7 | Organic sulfuric acids and derivatives | 266.15518 | 266.15501 | 14.9 | 16.3476079 | 0.061% |
| Ginsenoside F2 | C_42_H_72_O_13_ | 62025-49-4 | Prenol lipids | 784.49729 | 784.49805 | 11.301 | 14.9554148 | 0.056% |
| Guanosine | C_10_H_13_N_5_O_5_ | 118-00-3 | Purine nucleosides | 283.09167 | 283.09154 | 1.286 | 13.1538082 | 0.049% |
| 2-Aminobenzimidazole | C_7_H_7_N_3_ | 934-32-7 | Benzimidazoles | 133.064 | 133.06388 | 19.846 | 1.08385332 | 0.048% |
| Methoxytyrosine | C_10_H_13_NO_4_ | 7636-26-2 | Carboxylic acids and derivatives | 211.08446 | 211.08392 | 5.762 | 12.2882485 | 0.046% |
| 1-Isopropenyl-2,3-dihydro-1H-benzo[d]imidazol-2-one | C_10_H_10_N_2_O | 52099-72-6 | Benzimidazoles | 174.07931 | 174.0785 | 10.487 | 11.1768513 | 0.042% |
| 4-Pyridoxic acid | C_8_H_9_NO_4_ | 82-82-6 | Pyridines and derivatives | 183.05316 | 183.05366 | 18.651 | 9.71364522 | 0.036% |
| 2-Phenylacetamide | C_8_H_9_NO | 103-81-1 | Benzene and substituted derivatives | 135.06841 | 135.06848 | 1.232 | 0.76940533 | 0.034% |
| Tangeritin | C_20_H_20_O_7_ | 481-53-8 | Flavonoids | 372.1209 | 372.12078 | 11.629 | 0.74010679 | 0.033% |
| Crotamiton | C_13_H_17_NO | 483-63-6 | Benzene and substituted derivatives | 203.13101 | 203.13095 | 13.187 | 0.73990698 | 0.033% |
| Cetrimonium | C_19_H_41_N | 6899-10-1 | Organonitrogen compounds | 283.3239 | 283.32366 | 12.311 | 0.72997692 | 0.032% |
| Nobiletin | C_21_H_22_O_8_ | 478-01-3 | Flavonoids | 402.13147 | 402.13127 | 11.107 | 0.72284691 | 0.032% |
| Osthol | C_15_H_16_O_3_ | 484-12-8 | Coumarins and derivatives | 244.10994 | 244.10988 | 12.483 | 0.71527583 | 0.032% |
| 1-Linoleoyl glycerol | C_21_H_38_O_4_ | 2277-28-3 | Fatty Acyls | 354.27701 | 354.27684 | 12.12 | 0.709997 | 0.031% |
| 2-Amino-4-methylpyrimidine | C_5_H_7_N_3_ | 108-52-1 | Diazines | 109.064 | 109.06427 | 17.828 | 0.70053037 | 0.031% |
| Benzaldehyde | C_7_H_6_O | 100-52-7 | Benzene and substituted derivatives | 106.04186 | 106.04224 | 7.765 | 0.65494379 | 0.029% |
| 1-palmitoyl-2-hydroxy-sn-glycero-3-phosphoethanolamine | C_21_H_44_NO_7_P | 53862-35-4 | Glycerophospholipids | 453.28554 | 453.28533 | 12.659 | 0.60268582 | 0.027% |
| Senkyunolide A | C_12_H_16_O_2_ | 63038-10-8 | Isobenzofurans | 192.11503 | 192.11514 | 11.702 | 0.51493324 | 0.023% |
| Azelaic acid | C_9_H_16_O_4_ | 123-99-9 | Fatty Acyls | 188.10486 | 188.10403 | 7.472 | 6.03548221 | 0.022% |
| Hesperidin | C_28_H_34_O_15_ | 520-26-3 | Flavonoids | 610.18977 | 610.19026 | 7.29 | 5.68687303 | 0.021% |
| N,N'-Diphenylurea | C_13_H_12_N_2_O | 102-07-8 | Benzene and substituted derivatives | 212.09496 | 212.09485 | 10.812 | 0.43479455 | 0.019% |
| Oleic acid | C_18_H_34_O_2_ | 112-80-1 | Fatty Acyls | 282.25588 | 282.25572 | 18.401 | 5.14064693 | 0.019% |
| Rosmarinic acid | C_18_H_16_O_8_ | 20283-92-5 | Cinnamic acids and derivatives | 360.08452 | 360.08439 | 7.443 | 5.0372515 | 0.019% |
| Naringin | C_27_H_32_O_14_ | 10236-47-2 | Flavonoids | 580.17921 | 580.17946 | 7.046 | 4.72565663 | 0.018% |
| LPE 16:0 | C_21_H_44_NO_7_P | 13190-01-7 | Glycerophospholipids | 453.28554 | 453.2856 | 12.686 | 3.78117789 | 0.014% |
